# Rheumatico-Catarrhal Epidemic

**Published:** 1836

**Authors:** Henry Perrine

**Affiliations:** United States’ Consul


### Art. III.—Rheumatico-Catarrhal Epidemic.

*An account of an Epidemic in which symptoms of Catarrh and Rheumatism were combined, at Campeche, in the province of Yucatan, in the winter of* 1835–6. By Henry Perrine, M. D. United States' Consul.

TO PROFESSOR DRAKE, EDITOR OF THE WESTERN MEDICAL JOURNAL IN CINCINNATI, OHIO.

*Consulate, U. S. A. Campeche,* 1*st March,* 1836.

Sir,—As mild epidemics have, frequently, in the winter preceded the severe epidemic cholera called Asiatic, and as in the winter of 1833, a species of influenza pervaded this city, which was invaded by the terrible cholera of the succeeding summer, I have thought it advisable to write you a few lines concerning an almost universal epidemic of the present year. Towards the latter part of December it was frequently remarked, that the weather was warmer than usual for that month, and that, nevertheless, slight *catarrhs* were much more frequent. At the beginning of January, the cases became so numerous as to attract my attention, although few were so violently attacked, as to require medical assistance for the epidemic alone. On the 6th, at night, I myself felt a general disturbance, rheumatic pains in nearly all the moveable portions of my body, suppression of the secretions, slight heat of skin, alternating with chilly sensations, an unconquerable lethargy and absence of all appetite, which confined me two days and nights to my hammock, and two days and nights longer to my room. Abstinence was my principal remedy, laxatives and diluents were added, and opiate sudorifics calmed my pains, and completed the cure. As the epidemic had been so generally christened a catarrh, I was astonished that no catarrhal symptoms in the respiratory apparatus, had appeared in my case. Neither sneezing nor snuffling, nor coughing nor hawking, nor the flow of mucus from my nostrils, nor expectoration from my lungs, nor difficulty of breathing, nor pain in my breast. After recovery, upon inquiry and observation, I found that the great majority had been affected in a similar manner; that the soreness and pains of the muscles and joints, or the *break-bone* feeling of Dr. Rush, were the prominent, principal, and, indeed, the only essential symptoms. As, however, the catarrhal symptoms were also present, in a considerable number of cases, I contrived the compound epithet of the "*Rheumatic Catarrhal Epidemic*" to serve as a general name.

In some individuals, the affections were so slight, that they did not leave the streets. Others were content to pass one or two days in bed. But some again continued confined to their hammocks during five, six, or seven days; and others, though few in proportion to the whole number attacked, were finally carried to their graves. Of those whose lives were cut short, very few had applied for medical assistance, under the impression that domestic remedies were sufficient for their cure. From what I could learn, however, I should make the distinction which we hear so often in the mouths of operative surgeons—that the operation was not the *cause*, but solely the *occasion* of the death of their patients. I should, in like manner say, that the epidemic was not the cause, but the occasion of the deaths which occurred. In individuals predisposed to pulmonary disorders, the whole irritation of the epidemic became concentrated on the predisposed parts. In a lady under my care, it excited an attack of asthma, which became the principal object of treatment, and lasted fifteen days and nights, during the whole of which period she continued sitting bolstered in an arm chair.

In numerous cases, the individuals attacked by the Rheumatic-Catarrhal Epidemic, presented, *in a few days*, the symptoms of peripneumonia of pneumonia, of pleuritis separately, or of bronchitis, pneumonitis and pleuritis, all combined. My views of the nature of these affections, prevented my having recourse to general bleeding, but in consultations I had an opportunity of seeing its noxious results. One physician, who had bled six different patients, assured me that he could not find the buffy coat, nor any other indication of acute inflammation in the blood of either; that all grew worse, and that some died. In all cases, I have been forced to consider the local affections as the consequence, and not the cause, of the general affections. I have practised nearly seventeen years, in countries where intermittent and remittent fevers are endemic diseases. In the beginning, I used the lancet freely, whenever *signs* of local inflammations supervened; but my misfortunes in this mode of practice, very soon induced me to become much more cautious. I, nevertheless, for a long time, considered the local affections as accidental complications, instead of occasional results; and hence adopted the contradictory treatment of the books. Dr. Hosack's compound name of typhous-pneumonia, appeared to me significant enough of the disease to which he applied it; and the phrase bilious pneumonia was to me equally satisfactory.

I cited with satisfaction the observations of the great authors, "that during the prevalence of an epidemic, all other diseases wear its livery;" and believed that the same law might be discovered during the prevalence of endemic diseases. My observations, however, gradually led me to reason in a different manner. I saw that in many, very many cases, the endemic did wear the livery of many other diseases; that opthalmias, rheumatisms, diarrhœas, &c. wear the liveries of intermittents, and are cured by the bark or quinine alone. I saw symptoms of peripneumonia and pleuritis, of hepatitis and gastritis, appearing in remittent fevers, hours and days after the general disturbance, and disappearing hours and days both before and after the febrile symptoms disappeared; and I moreover saw, that, in such cases, both the local and general symptoms were relieved or removed much more promptly by the aggregated prescriptions of large doses of quinine, than by any other method.

As I became bolder in my practice, I found, that by cutting short the general febrile symptoms with large doses of quinine, the signs of local inflammation did not proceed beyond their incipient stages; and finally I became convinced, that in all indications of local inflammation *resulting* from the general fever, the antiphlogistic treatment for the indisputable phlegmasia, was not only unnecessary, but frequently very injurious. I found that, in some years and some seasons, the endemic fevers presented congestive or inflammatory symptoms in the breast, and during others, that they appeared oftener in the viscera of the abdomen. I varied, therefore, my nomenclature, so as to fix the attention primarily on the general fever, and secondarily, on the local affections which resulted from it. Instead of pneumonia typhoidia, I suggested pneumonic typhus; and in those cases of the same epidemic typhus, where the determination to the head was the most striking symptom, I should have affixed the epithet phrenitic. I reflected, that the popular term "pleurisy in the head," indicated a fever with determination both to the lungs and brain, either mediate or intermediate. So with our autumnal remittents, commonly called bilious pneumonias. The term peripneumonic remittent was to me most satisfactory, when the mucous surface of the lungs was the principal sufferer, and hepatic remittent when the liver bore the greatest share.

This tedious digression, in this off-hand sketch, is not so foreign to the subject of my letter, as at first sight may appear. The rheumatic catarrhal epidemic of Campeche, wore the livery of various phlegmasiæ; but antiphlogistic remedies to such an extent as would be required by true local inflammations, with symptomatic fevers, I repeat were not only unnecessary, but injurious.

The natural crisis of the epidemic was almost universally a general perspiration. As I have said before, a great majority got well without medical aid, by abstinence, rest, and diluents. Their lemonades were taken cool, while the skin was dry and warm; and tepid after diaphoresis began. Even leeches, scarifications, or cuppings, were rarely necessary even in the worst cases of local congestion. The pain in the lungs or liver was not accompanied either by that dryness, redness, or furriness of the tongue, or strength, frequency, or hardness of the pulse, which is found in true pneumonits or hepatitis. The difficulty in respiration, and painful cough, by some considered symptoms of positive inflammation of the lungs or liver, generally depended on the state of the intercostal or abdominal muscles. I have had four Broussaist physicians dispute with me, in a consultation, the existence of acute inflammations, founded on the two symptoms of cough and pain alone. All the secretions were free; bowels loose; urine free; tongue clean and moist; skin cool and perspiring; pulse slow, soft and full; appetite and sleep good; the woman, in fact, convalescing—but the pain! the pain! *augmented by pressure!!* the difficulty in taking a full respiration!! Fifty leeches at least, and a large epispastic must be applied!

The most important intelligence concerning any and all forms of diease, consists in the most successful mode of treatment. However formidable may be the symptoms, if a simple combination of well known remedies soon puts them to flight, it is idle to enter into details of the numerous forms of epidemic or endemic diseases. Whatever has been the livery which this epidemic has put on, the universal rheumatic pains have always betrayed it. Whether under the form of pectoral or abdominal inflammation, or congestions, with or without cough and difficulty of respiration, this soreness of the muscles, stiffness of the joints—in short, this general breakbone feeling, such as is experienced by a person the day after excessive exertion of the whole body, showed the true character of the disease.

It is said that at least three-fourths of the whole population of Campeche have suffered it, and that it is extending through the province with more or less mortality. In one family, seventeen persons, including the servants, were all down on the same day. In many instances entire families have been obliged to depend on the charity of other families, for the preparation even of sick food; and the convalescents of that family, have repaid the services to their friends under the same calamity.

My most general and effective *medical* prescription, consisted of the following composition for adults: one-sixth to one-fourth grain acet. morphin: one-half to one grain of ipecacuana, three to four grains of pil. hydrargyri, made into one pill. This dose of *one* pill to be repeated every hour or every two hours, according to the intensity of the pains or cough, until relieved; and to be continued, at intervals of every three or four hours afterwards, until all the uncomfortable feelings disappear. Generally after the second dose, the pains became relieved, and a free sweat appeared. As soon as the sweat began to dry, or the pains to return, say in three or four hours, another pill was given, and followed by relief of pain, and restoration of perspiration. Acidulated drinks, as lemonade, &c. were taken, ad libitum—the only restriction being to drink them tepid, while the sweat was present.

Diet, of course gruels, arrow root, &c. although the prejudice here, in favor of broths and soups, is so inveterate, that it is likely my prohibition of them was disregarded.

The success, and consequent popularity of this compound pill, has been proportionately as great as my large doses of quinine, (10 grains) in intermittents, and my large dose of calomel, (40 grains) in cholera. I doubt whether the famous powder of James, or of Dover, have ever had greater reputation as a sudorific. As an anodyne sudorific, it certainly merited the applauses of the sufferers, in this painful epidemic. The blue pill I consider an essential ingredient, for which the sulphate of potash, in Dover's powder, is not by any means an equivalent. While opening the secretions of the liver, and thus sympathetically those of the skin, the flow of bile thus promoted, serves as a laxative, which counteracts the torpifying effects of the morphine on the peristaltic action of the intestines. In short, this composition opens all the secretions of the body more readily, and more effectually, than any of the analogous combinations of the materia medica which I have tried. An apothecary has commenced the practice of medicine here, with some success and more reputation, whose principal stock of knowledge and of medicine consists in these anodyne, sudorific, laxative pills!!

HENRY PERRINE.

